# 

**Published:** 1896-07-25

**Authors:** 


**Notices of Births, Deaths, and Marriages** are inserted in this column at a charge of 2s. 6d. for an announcement not exceeding four lines.

### NOTICE TO CORRESPONDENTS.

All MS., Letters, Books for Review, and other matters intended for the Editor should be addressed The Editor, The Lodge, Porchester Square, London, W.

The Editor cannot undertake to return rejected MS., even when accompanied by stamped directed envelope.

**Notice to Advertisers and Subscribers.**

All Advertisements, Orders for Copies of The Hospital and Business Communications should be addressed to **THE MANAGER, (Not to The Editor)**, The Hospital, 428, Strand, London, W.C.

The Cost of Subscription, post free, is as follows:—Per week, 2½d.; per quarter, 2s. 8d.; per half-year, 5s. 3d.; per annum, 10s. 6d. Foreign:—Per Annum, 15s. 2d.; payable, in all cases, in advance.

Just Published.

**"Burdett's Hospitals and Charities, 1896."**

Seventh year of publication. Over 1,000 pp. Scarlet cloth, gilt lettered. Price 5s. A most complete guide to British, Colonial, Indian, and American Hospitals, Dispensaries, Nursing and Convalescent Institutions, and Asylums. A few complete sets of the preceding six issues of the "Annual" may still be obtained on application to the Publishers, The Scientific Press (Limited), 428, Strand, London, W.C.

**NOTICE.—Vol. XIX. of "The Hospital" (October, 1895, to April, 1896), in handsome cloth binding, is now on sale at the Office of this Journal. Price 7s. 6d. Cases for binding the Numbers contained in this Volume. 1s. 6d. Index to Vol. XIX., 2½d., post free. The Monthly Part for August will be ready on August 1st, price 6d., by post 8d.**

### Books Received.

A. and C. Black.

"Rheumatism: Its Nature, its Pathology, and its Successful Treatment." By T. J. Maclagan, M.D.

John Wright and Co.

"A Text-Book of Histology." By Arthur Clarkson, M.B., C.M.Edin.
"The Bacteriological Examination of Water for the Typhoid Bacillus." By T. H. Pearmain and C. G. Moor. Reprinted from "The Analyst," May—June, 1896.

### Scraps and Gleanings.

A cottage fever hospital is to be built at Bridge of Alford, near Aberdeen.

The East Suffolk Hospital has received a donation of £100 from Captain Pretyman, M.P.

The plans of the new buildings of the Bedford General Infirmary have been chosen.

The Beccles Hospital spent £545 during the past year, and there is now a small balance of £13 against the hospital.

In the new isolation hospital at Budapest there is a section devoted to the treatment of patients bitten by dogs, wolves, or other rabid animals.

The Princess Mary Village Homes at Addlestone, Surrey, are appealing for funds to build a convalescent cottage. About £700 is required.

Mr. H. Littlewood, F.R.C.S., one of the honorary assistant surgeons at the General Infirmary, Leeds, has been appointed one of the honorary surgeons of the institution.

The Hygienic Lighting and Heating Company have obtained the gold medal for the best safety lamp at the Life Saving Exhibition recently held at the Central Hall, Holborn.

In the Eppendorf Hospital at Hamburg, the largest modern hospital in Europe, there were no fewer than 3,000 cholera patients at one time during the cholera epidemic in 1892.

The receipts of the Romford Cottage Hospital for the past year amounted to £407 and the expenses to £391. Seventy-four patients were treated in the wards during the year.

Only one legacy—one of £17—was received by the Yarmouth Hospital during the past year. Annual subscriptions and church and he Saturday collections showed an increase.

The balance-sheet of the Dundee Hospital Saturday Committee shows receipts amounting to £161 and expenses amounting to £12, leaving a balance of £149, which has been handed over to the Royal Infirmary.

Belfast Hospital Saturday is not a flourishing institution. The amount collected in 1884, when the Fund was inaugurated, viz., £530, has never since been equalled, and the result this year is not one-half of that amount.

The recent fête in Hanworth Park on behalf of Guy's Hospital Fund realised close upon £1,200. The contributions and donations towards the New Convalescent Home for the Middlesex Hospital amount to upwards of £4,000.

The National Eye and Ear Infirmary, Dublin, received 391 in-patients and 2,791 new out-patients during 1895. The joint committee of this hospital and St. Mark's Ophthalmic Hospital are now engaged in the collection of the fund required for the building of the amalgamated hospital.

The total number of in-patients treated during the year ended April 1st, 1896, in the eleven hospitals under the supervision of the Board of Superintendence of Dublin Hospitals amounted to 10,508. The time spent in hospital by each patient under treatment (omitting the patients in the Royal Hospital for Incurables) averaged 22·76 days.

The Darlington Children's Hospital, a small institution with seven beds, which was for 25 years supported entirely by Miss Emma Gurney Pease, has, under the terms of her will, been closed. The work, however, is to be carried on at the Darlington Hospital and Dispensary, to which a wing is being added in order to provide the extra accommodation necessary.

As a rule there are thirty or thirty five poor women waiting to be admitted as in-patients to the New Hospital for Women in Euston Road. At present there is rarely a bed available at once for an urgent case, nor is there any means of isolating a case of infectious illness in the hospital. Want of funds prevents the committee from increasing the number of beds.

As the result of a visit to over twenty fever hospitals on the Continent, Bailie Pollard, chairman of the Public Health Committee of the Edinburgh Town Council, gives it as his opinion that Prague is at the present time the most insanitary capital north of the Alps, and in mortality from small-pox it can only be compared with the still neglected rural districts of Hungary.

The "After-Care Association for Poor Persons Discharged from Asylums for the Insane"—to give it its full title—during the year 1895 assisted over 100 cases by boarding them out in cottages, making them grants of money or clothing, or finding them occupation. The income for the year amounted to £541, of which about one quarter (£152) was contributed by the friends of applicants, and the expenditure to £452.

The Birmingham Hospital Saturday Fund are at last able to supplement the work done for adults at their convalescent homes at Llandudno, by the addition of a home for children. A house called Bryn Marle, situated midway between the two present homes, has been taken for the purpose by a gentleman, who prefers to remain anonymous. He has undertaken to furnish it and to bear the entire cost of maintenance for 12 months.

The annual report of the Leyton, Walthamstow, and Wanstead Children's and General Hospital, for the first complete year since the enlargement of the hospital in 1894, shows that 263 in-patients and 100 minor accident and casualty cases were treated. The average weekly cost per bed was £1 0s. 10d. The income (including £2,618 bequests and donations) amounted to £4,512, and the expenditure (including £631 for improvements to and furnishing "The Homecroft") to £4,309.

Four hundred and ninety women were treated in the wards of the City of London Lying-in Hospital in 1895, and 1,585 were delivered at their own homes. Both these numbers show a slight decrease compared with the previous year's figures. Forty-one midwives and 160 monthly nurses were trained at the hospital during 1895. Of the midwives 32 presented themselves for and passed the London Obstetrical Society's examination. The receipts for the year amounted to £4,038, and the expenditure to £3,716. The latter item includes £92 spent on extraordinary repairs and improvements.

### The Student of Medicine.

### APPOINTMENTS.

Dr. Annie M. S. Anderson, appointed Honorary Assistant Physician to the Clinical Hospital for Women and Children, Manchester. A. M. Archer, B.A., M.D., appointed Medical Officer to the Chester Post Office. F. F. Burman, L.R.C.P.Edin., M.R.C.S., appointed Medical Officer of Health to the Wath District Council. H. Campbell, M.D.Lond., F.R.C.P., appointed Physician to the West End Hospital for Diseases of the Nervous System, Paralysis, and Epilepsy, Welbeck Street, W. F. Chown, M.B.Lond., L.S.A., appointed Medical Officer for the Fourth District of the Helston Union. H. Colbatch Clark, M.R.C.S., L.R.C.P.., appointed Junior House Physician to the North-Eastern Hospital for Children, Hackney Road. J. E. P. Davies, M.B., B.Sc.Lond., M.R.C.S., L.R.C.P., appointed House Physician to St. Mary's Hospital. D. J. Graham, M.B., C.M.Edin., appointed Resident Medical Officer to Chalmers' Hospital, Edinburgh. H. E. Haymes, M.R.C.S., L.R.C.P., appointed Assistant Medical Officer to the Royal Berks Hospital, Reading. T. M. Legge, M.A., M.D., B.Ch.Oxon., D.P.H.Camb., appointed Professor of Hygiene to the Bedford College for Women, London. H. Littlewood, F.R.C.S., Honorary Assistant-Surgeon, appointed Honorary Surgeon of the General Infirmary, Leeds. G. B. Millett, L.R.C.P.Edin., M.R.C.S., appointed Medical Officer of Health to the] West Penwith Port Sanitary Authority. Dr. O'Rourke, appointed Medical Officer for the Ballyconnell Dispensary District of the Bawnboy Union. H. W. Pepper, M.R.C.S., L.R.C.P., appointed House-Surgeon to Royal Berks Hospital, Reading. H. Reeks, M.R.C.S., L.R.C.P.Lond., appointed Medical Officer for the Workhouse of the Steyning Union. S. H. Rentzsch, L.R.C.P.Lond., M.R.C.S.Eng., appointed Public Vaccinator for the Cheshunt District of the Edmonton Union. G. H. Ruston-Harrison, M.B., C.M.Edin., appointed House-Physician to the Royal Berks Hospital, Reading. B. Saunders, M.B., M.C.Aberd., appointed House-Physician to the City of London Hospital for Diseases of the Chest, Victoria Park, E. Mr. C. L. B. Stares, appointed Assistant Medical Officer to the Wandsworth and Clapham Union Infirmary. Mr. F. G. Wade, appointed Medical Officer of Health to the Cricklade and Wootton Bassett Rural District Council, and Medical Officer for the First District of the Cricklade and Wootton Bassett Union. E. Seaton, M.D.Lond., M.R.C.S., appointed Examiner for Diploma in Public Health of the Royal College of Surgeons of England. T. W. Shore, M.D., B.Sc.Lond., M.R.C.S.Eng., appointed Examiner in Elementary Biology for the First Examination of the Royal College of Surgeons of England. W. G. Spencer, M.B.Lond., F.R.C.S.Eng., appointed Examiner in Physiology for the Second Examination of the Royal College of Surgeons of England. E. H. Starling, M.B., B.S.Lond., M.R.C.S., appointed Examiner in Physiology for the Fellowship of the Royal College of Surgeons of England. T. G. Stevens, M.D.Lond., F.R.C.S.Eng., appointed Examiner in Elementary Biology for the First Examination of the Royal College of Surgeons of England. W. Stirling, M.D., B.Sc., F.R.S., appointed Examiner in Physiology for the Fellowship of the Royal College of Surgeons of England. J. B. Sutton, F.R.C.S.,Eng., appointed Examiner in Anatomy for the Fellowship of the Royal College of Surgeons of England. G. Vawdrey, L.R.C.P.Edin.. M.R.C.S.Eng., appointed Medical Officer of Health to the Farnborough Urban District Council.

### VACANCIES.

The following vacancies are announced this week, the amount of salary in each case being given after the name of the institution:—

### Resident Medical Officers.

City of London Hospital for Diseases of the Chest, Victoria Park, E.—Resident Medical Officer. Salary £100 per annum, with board, &c. Must be doubly qualified. Applications to the Secretary at the hospital by July 31st.

Denbighshire Infirmary, Denbigh.—House-Surgeon; doubly qualified and conversant with the Welsh language. Salary £80 per annum, with board, residence, and washing. Applications to W. Vaughan Jones, Secretary, by July 30th.

General Hospital, Nottingham.—House-Surgeon. Salary £100 per annum, rising £10 a year to £120. Applications to the Secretary by August 8th.

Nottingham Borough Asylum, Mapperley Hill, Nottingham.—Second Assistant Medical Officer, unmarried. Salary £100 per annum, with apartments, board, washing, &c. Applications to the Medical Superintendent by July 27th.

Royal Eye Hospital or Royal South London Ophthalmic Hospital, Southwark.—House Surgeon; will be required to take up residence about October 1st. Salary £50 per annum, with board and lodging. Applications to the Secretary by August 1st.

Sussex County Hospital, Brighton.—Fourth Resident Medical Officer; doubly qualified, unmarried, and under 30 years of age. Salary £30 per annum, with board, washing, and residence in the hospital. Applications to the Secretary by August 5th.

Wolverhampton and Staffordshire General Hospital, Wolverhampton.—Resident Assistant. Appointment for six months. Board, lodging, and washing provided. Applications, inscribed "Application for Resident Assistant," to be addressed to the Chairman of the Medical Committee by July 27th.

### Non-Resident Medical Officers.

Glasgow Samaritan Hospital for Women, Victoria Road, Glasgow.—Clinical Assistant (Lady). Applications to Thos. Macquaker, Secretary, 89, West Regent Street, Glasgow, by July 25th.

Owens College, Manchester.—Assistant Lecturer and Demonstrator in Materia Medica and Pharmacy. Stipend £120 per annum. Applications to the Registrar by July 31st.

